# Constitutive Activation of NF-κB Pathway in Hematopoietic Stem Cells Causes Loss of Quiescence and Deregulated Transcription Factor Networks

**DOI:** 10.3389/fcell.2018.00143

**Published:** 2018-10-30

**Authors:** Masahiro Marshall Nakagawa, Huanwen Chen, Chozha Vendan Rathinam

**Affiliations:** ^1^Department of Genetics and Development, Columbia University Medical Center, New York, NY, United States; ^2^Institute of Human Virology, Baltimore, MD, United States; ^3^Center for Stem Cell & Regenerative Medicine, Baltimore, MD, United States; ^4^Marlene & Stewart Greenebaum Comprehensive Cancer Center, School of Medicine, University of Maryland, Baltimore, MD, United States

**Keywords:** hematopoietic stem cells, NF-κB, quiescence, transcription factors, signal transduction

## Abstract

Identifying physiological roles of specific signaling pathways that regulate hematopoietic stem cell (HSC) functions may lead to new treatment strategies and therapeutic interventions for hematologic disorders. Here, we provide genetic evidence that constitutive activation of NF-κB in HSCs results in reduced pool size, repopulation capacities, and quiescence of HSCs. Global transcriptional profiling and bioinformatics studies identified loss of ‘stemness’ and ‘quiescence’ signatures in HSCs with deregulated NF-κB activation. In particular, gene set enrichment analysis identified upregulation of cyclin dependent kinase- *Ccnd1* and down regulation of cyclin dependent kinase inhibitor *p57^kip2^*. Interestingly, constitutive activation of NF-κB is sufficient to alter the regulatory circuits of transcription factors (TFs) that are critical to HSC self-renewal and functions. Molecular studies identified *Junb*, as one of the direct targets of NF-κB in hematopoietic cells. In essence, these studies demonstrate that aberrant activation of NF-κB signals impairs HSC quiescence and functions and alters the ‘TF networks’ in HSCs.

## Introduction

Hematopoiesis is a process through which all the blood cells are generated through sequential cell divisions and differentiation of progeny that originate from hematopoietic stem cells (HSCs) (Kondo et al., 2003). Under steady state conditions HSCs remain largely quiescent (Morrison and Weissman, 1994; Passegue et al., 2005), and divide < 20 times in their entire life span (Wilson et al., 2008; Trumpp et al., 2010). A tight control on quiescence is vital for maintaining the “stemness” of HSCs (Wilson and Trumpp, 2006; Wilson et al., 2008). Indeed, decision of HSCs to remain at a quiescent state or enter into an actively proliferating state is controlled by number of factors through both cell intrinsic and extrinsic mechanisms. In response to extrinsic soluble factors; inflammatory cytokines such as interferon (IFN)-α and IFN-γ; growth factors such as granulocyte colony stimulating factor (GCSF), stem cell factor (SCF), and thrombopoietin (TPO);cytokines such as transforming growth factor (TGF) -β and tumor necrosis factor (TNF)-α; and chemokines such as the stromal cell derived factor (SDF)-1, HSCs can either enter dormancy or cell cycle (Wilson and Trumpp, 2006; Kiel and Morrison, 2008; Trumpp et al., 2010). Intrinsic factors that regulate HSC quiescence includes; cell cycle inhibitors such as p21 and p57; transcription factors (TFs) such as Gfi1, Egr1, FOXOs, and PBX1; and ubiquitin ligases such as c-Cbl, Itch, Fbxw7, and A20 (Orford and Scadden, 2008; Rathinam et al., 2008, 2011; Thompson et al., 2008; Trumpp et al., 2010; Zou et al., 2011; Nakagawa et al., 2015). A harmony between intrinsic and extrinsic factors is essential for proper maintenance of HSCs in the bone marrow niche.

Nuclear factor kappa-light-chain-enhancer of activated B cells (NF-κB) proteins act as TFs and are regarded as the master regulators of innate and adaptive immunity (Vallabhapurapu and Karin, 2009). They are five key members of this family in mammals: Rel A (p65), Rel B, c-Rel, p50/p105 (also known as NF-κB1) and p100/52 (also known as NF-κB2) (Ghosh and Hayden, 2008). NF-κB signals are activated in response to various upstream stimuli. In the absence of any activating signals, inhibitor of NF-κB (IκB) proteins form complexes with inactive NF-κB proteins and remain in the cytoplasm. Nevertheless, following an activating signal, the IκB kinase (IKK) complex, which is composed of two kinases; IKK1 (IKKα), IKK2 (IKKβ) and a regulatory subunit NEMO (IKKγ), phosphorylates IκB, which leads to ubiquitylation and subsequent degradation of IκB (Rothwarf et al., 1998). This results in the release of NF-κB complexes from the cytoplasm to the nucleus, where they activate expression of target genes (Ghosh and Hayden, 2008). Therefore, in the entire cascade of NF-κB signals IKK complex plays a major role, as decontrolled activation of the IKK complex can lead to detrimental downstream consequences. IKK complex phosphorylates IκB proteins at two amino (N)-terminal regulatory serine residues (Brown et al., 1995). In majority of the canonical NF-κB signaling pathways, IKK2 is necessary and sufficient to phosphorylate IκB and activate NF-κB (Ghosh and Hayden, 2008).

Inflammatory control of HSCs has emerged as a novel mode of HSC regulation in the recent years. HSCs are believed to sense immune insults by both cell intrinsic, and extrinsic mechanisms (Welner et al., 2008; Baldridge et al., 2011; King and Goodell, 2011). Similar to differentiated immune cells, HSCs recognize pathogens through their expression of toll-like receptors (TLRs). Ligation of TLR signals in HSCs leads to proliferation and differentiation (Nagai et al., 2006; Takizawa et al., 2017). On the other hand, cell extrinsic mode of recognition of an immune insult by HSCs involves signaling through receptors for pro-inflammatory cytokines. A spectrum of pro-inflammatory cytokines and chemokines, that includes IL1, IL6, IL8, TNF, CC-Chemokine ligand 2 (CCL2), IFN-α and IFN-γ (Baldridge et al., 2011; King and Goodell, 2011; Mirantes et al., 2014) has been shown to influence HSCs. Of note, prolonged exposure of HSCs to pro-inflammatory cytokines causes diminished self-renewal and quiescence (Essers et al., 2009; Sato et al., 2009; Baldridge et al., 2010). In this regard, NF-κ B can be viewed as a gatekeeper on the inflammatory control of HSCs. Indeed, NF-κ B is the major downstream effector of signals transduced by both TLRs and pro-inflammatory cytokines (Ghosh and Hayden, 2008; Vallabhapurapu and Karin, 2009). In addition, uncontrolled NF-κ B activity results in increased expression of pro-inflammatory cytokines, including TNF, IL1, IL6, and IFN-γ (Boone et al., 2004; Coornaert et al., 2009; Dong et al., 2010; Harhaj and Dixit, 2012). A recent study from our group demonstrated that defects in the negative regulatory circuits of NF-κ B signaling cause loss of quiescence and pre-mature depletion of HSCs (Nakagawa et al., 2015). While these studies highlight the importance of NF-κ B pathways in HSC quiescence, the downstream consequences of NF-κ B signals and the precise mechanisms through which NF-κ B controls HSCs remain largely unknown. More importantly, constitutive activation of NF-κB has been documented in different types of human diseases, including myeloid neoplasms (Brown et al., 1995; Guzman et al., 2001; Panagopoulos et al., 2013). In particular, NF-κB has been shown to be constitutively active in leukemic stem cells (LSCs) (Kagoya et al., 2014). These links to human disease provide a compelling rationale for more detailed investigations into the mechanisms through which NF-κB induces leukemogenesis and transformation of normal HSCs to LSCs. Moreover, these studies were conducted with LSCs and the consequences of constitutive NF-κB signaling in normal HSCs remains unexplored.

Thus, in the present study we attempted to define the role of NF-κB in HSC biology and to gain molecular insights into mechanisms through which NF-κB impacts HSCs. To this end, we followed a ‘gain of function’ approach to delineate NF-κB functions in HSCs. Using mice that express the constitutively active form of IKK2 in HSCs, we show that the HSC pool was reduced and HSC functions were compromised. HSCs from these mice exhibited a hyper-proliferative phenotype associated with loss of quiescence. Genome-wide transcriptional profiling studies combined with ‘*in silico*’ analysis identified deregulated molecular and genetic signatures of HSCs and ‘TF network’ in HSCs with constitutive activation of NF-κB. Furthermore, molecular studies identified deregulated expression of the quiescence factor-*p57* and that *Junb* as one of the key targets of NF-κB in hematopoietic cells. Taken together, these data indicate that NF-κB signaling plays a key role in the determination of ‘quiescence’ vs. ‘active’ state of HSCs and that fine-tuning of NF-κB signaling preserves the molecular and genetic identities of HSCs.

## Materials and Methods

### Mice

R26STOPFLIKK2ca (B6-Gt(ROSA)26Sortm1(Ikbkb)Rsky/J Stock #: 008242) transgenic mice (Sasaki et al., 2006) and Vav-Cre(B6.Cg-Commd10Tg(Vav1-icre)A2Kio/J, stock #: 008610) (de Boer et al., 2003) mice were purchased from the Jackson laboratory. B6.CD45.1 congenic (stock #: 002014) congenic animals were purchased from the National Cancer Institute. All mouse experiments were approved by the Institutional Animal Care and Use Committee (IACUC) of Columbia University and University of Maryland School of Medicine.

### Bone Marrow Transplantation

1 × 10^6^ of bone marrow cells were injected into lethally irradiated (10 Gy) congenic (CD45.1^+^) recipient mice. For competitive-repopulation experiments, 5 × 10^5^ of bone marrow cells were mixed with equal numbers of CD45.1^+^ competitor cells and injected into congenic recipients.

### Cell Proliferation and Quiescence

For bromodeoxyuridine (BrdU) assay, 3.33 mg of BrdU (BD Pharmingen) was injected intraperitoneally and mice were maintained on 0.8 mg/ml BrdU in the drinking water. After 16 h of injection, mice were sacrificed and bone marrow cells were stained for BrdU, following the BrdU flow kit manufacturer’s instructions (BD Pharmingen).

### Cell Cycle

For pyronin Y staining, cells were first incubated with 5 μg/ml hoechst 33342 (Life technologies) at 37°C for 45 min and then with pyronin Y (Sigma-Aldrich), at 1 μg/ml, for an additional 45 min at 37°C (Cheng et al., 2000). For side population assays, cells were incubated with 5 μg/ml Hoechst 33342 (Life Technologies) at 37°C for 90 min.

### Flow Cytometry

Cells were analyzed by flow cytometry with FACS Fortessa or LSR II (BD) and FACSDiva software (BD Biosciences) or FlowJo software (Tree Star). The following monoclonal antibodies were used: anti- CD34 (RAM34), anti-CD45.1 (A20), anti-CD45.2 (104), anti-CD48 (HM48-1), anti-CD117 (2B8), anti-Flt3 (A2F10.1), and anti-Sca-1 (D7) from BD Biosciences; anti-CD150 (TC15- 12F12.2) from Biolegend; anti-CD16/32 (93) and anti-CD127 (A7R34) from eBioscience. In all the FACS plots, indicated are the percentages (%) of the gated fraction.

### Apoptosis Assay

Apoptotic cells were detected by annexin V PE apoptosis detection kit according to the manufacturer’s instructions (BD Bioscience).

### Western Blot Analysis

Cells were lysed with cell lysis buffer (cell signaling) in the presence of protease inhibitor cocktail (complete, Roche) and 1 mM phenylmethylsulfonyl fluoride (PMSF) (Santa Cruz Biotechnologies). Cell lysates were subjected to 10% SDS-PAGE, transferred to PVDF membranes (Bio-Rad) and were treated with primary and secondary antibodies, respectively. The blots were visualized using the protoglow ECL (National Diagnostics) and image station 440 (Kodak). Antibodies used were as follows: anti- IκBα (44D4; Cell Signaling), anti-phospho- IκBα (5A5; Cell Signaling), anti-actin (I-19; Santa Cruz Biotechnologies), HRP-conjugated anti-mouse IgG (Cell Signaling), HRP-conjugated anti-rabbit IgG (Cell Signaling), and HRP-conjugated anti-goat IgG (Santa Cruz Biotechnologies).

### RNA Extraction and Real-Time PCR

Total RNA was isolated with RNeasy mini kit (Qiagen), then cDNA was synthesized with oligo (dT) primer and maxima reverse transcriptase (thermo scientific). Real-time PCR was performed in duplicates with a CFX-connect real-time PCR system (Biorad) and SsoAdvanced SYBR green supermix according to the manufacturer’s instructions (BioRad). Relative expression was normalized to the expression levels of the internal control-HPRT.

### ChIP Assay

Chromatin immunoprecipitation (ChIP) assay was performed with pierce agarose ChIP kit (Pierce) according to the manufacturer’s instructions. In brief, 1 × 10^7^ of bone marrow cells were fixed and immunoprecipitated with anti-p65 antibody (D14E12; Cell Signaling) or rabbit IgG (Pierce). Immunoprecipitated DNA fragment were quantified by real-time PCR with the use of the following primers, which amplify the *Junb* enhancer region containing NF-κB binding sites; forward 5′-ATAAGGTTCAGTACAAACGCCC-3′, reverse 5′-GCGTCACTGAGCTGAATAGG-3′. Fold enrichment was normalized to rabbit IgG-precipitated samples.

### Microarray

Total RNA of CD150^+^CD48^-^LSK cells from either control or IKK2^CA^ mice were isolated using the Qiagen RNAeasy micro kit according to the manufacturer’s instruction (Qiagen). Expression profiling was performed using Illumina’s MouseWG-6 v2.0 Expression BeadChip at Yale center for genome analysis. Normalized expression data were collapsed to gene symbols with max probes by collapsedataset module in Genepattern (Reich et al., 2006). These genes were pre ranked for log2 fold change and analyzed with gene set enrichment analysis (GSEA) (Mootha et al., 2003; Subramanian et al., 2005).

The list of TFs was selected with GO0003700 (sequence-specific DNA binding TF activity) from Quickgo with its default behavior (is_a, part_of, and occurs_in relationships only) (Binns et al., 2009).

### GO Analysis

Upregulated genes (more than 1.5 fold) or downregulated genes (less than 0.67 fold) in IKK2^CA^ mice were analyzed with DAVID 6.7 tools (Huang da et al., 2009).

### Statistical Analysis

Data represent mean ± SEM. Two-tailed Student’s *t*-tests were used to assess statistical significance (^∗^*P* < 0.05, ^∗∗^*P* < 0.01, ^∗∗∗^*P* < 0.001, and ^∗∗∗∗^*P* < 0.0001). For survival curve analysis, log rank test was used to assess statistical significance (^∗^*P* < 0.05 and ^∗∗^*P* < 0.01).

## Results

### Generation and Characterization of Mice With Constitutively Active IKK2 in HSCs

To investigate the gain of function role of NF-κB signals in hematopoiesis, we made use of the already available R26Stop^FL^ikk2^ca^ transgenic mice that were previously engineered (Sasaki et al., 2006) to express two serine to glutamate substitutions in the activation loop of the kinase domain of IKK2, preceded by a loxP-flanked STOP cassette, into the ubiquitously expressed ROSA26 locus and followed by a *frt*-flanked IRES-EGFP cassette (Sasaki et al., 2006). IKK2 containing these serine to glutamate mutations (IKK2^CA^) is constitutively active (Mercurio et al., 1997). We crossed the R26Stop^FL^ikk2^ca^ mice with the transgenic mice that express Cre under *Vav1* promoter, to obtain heterozygous IKK2^CA^Vav^cre^ (henceforth referred as IKK2^CA^) mice. *Vav1* promoter has been proven to be largely specific to the hematopoietic lineage, in particular, it is expressed at all stages of hematopoiesis, i.e., from HSCs to terminally differentiated myeloid, erythroid, and lymphoid lineage cells (de Boer et al., 2003).

**FIGURE 1:**
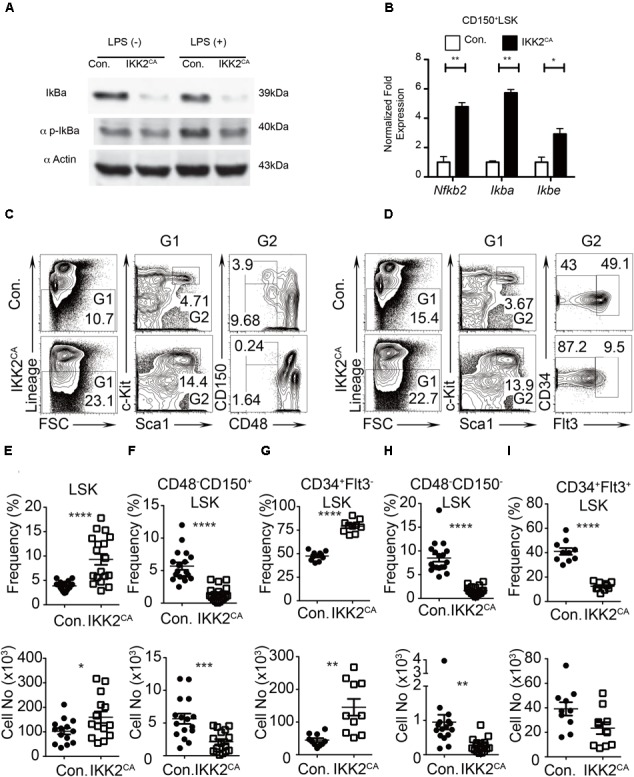
Reduced LT-HSC pool in IKK2^CA^ mice. **(A)** Western blot analysis of total IκBα protein and phospho-IκBα protein in splenocytes, from 6 weeks old IKK2^CA^ and control mice, either in the presence or absence of LPS stimulation for 1 h. Data are representative of two independent experiments. **(B)** Real time PCR analysis of NF-κB target genes in HSCs in the BM of 6 weeks old IKK2^CA^ and control mice. Data are representative of two independent experiments. **(C)** FACS plots of LSK cells, LT-HSCs (CD48^-^CD150^+^LSK), and MPPs (CD48^-^CD150^-^LSK) from the BM (two femurs and two tibias) of 6 weeks old IKK2^CA^ and control mice. Data are representative of five independent experiments. **(D)** FACS plots of LSK cells, ST-HSCs (CD34^+^Flt3^-^LSK), and MPPs (CD34^+^Flt3^+^LSK) from the BM (two femurs and two tibias) of 6 weeks old IKK2^CA^ and control mice. Data are representative of five independent experiments. Relative (top) and absolute (bottom) numbers of LSK (*n* = 14) **(E)**, LT-HSCs (CD48^-^CD150^+^LSK) (*n* = 16) **(F)**, ST-HSCs (CD34^+^Flt3^-^LSK) (*n* = 10) **(G)**, SLAM-MPPs (CD48^-^CD150^-^LSK) (*n* = 16) **(H)** and MPPs (CD34^+^Flt3^+^LSK) (*n* = 10) **(I)** in the BM (two femurs and two tibias) of 6 weeks old IKK2^CA^ and control mice. All data represent mean ± SEM. Two-tailed Student’s *t*-tests were used to assess statistical significance (^∗^*P* < 0.05, ^∗∗^*P* < 0.01, ^∗∗∗^*P* < 0.001, ^∗∗∗∗^*P* < 0.0001).

To directly asses if NF-κB is constitutively activated in IKK2^CA^ mice, we measured both total and phosphorylation levels of IκBα protein in hematopoietic cells. Western blot data indicated reduced levels of total IκBα protein and increased levels of phosphorylation IκBα protein in hematopoietic cells of IKK2^CA^ mice, even in the absence of any specific stimulation (Figure 1A). As expected, data of real-time PCR assays indicated increased expression levels of key NF-κB targets, such as *Nf-*κ*b2* (∼4.5 fold), *I*κ*Bα* (∼ 6 fold), and *I*κ*B𝜀* (∼ 3.5 fold) in IKK2^CA^ CD150 ^+^CD48^-^LSK cells (Figure 1B). These data suggest that NF-κB signaling is constitutively active in the HSCs of IKK2^CA^ mice.

### Diminished LT-HSC Pool in the BM of IKK2^CA^ Mice

IKK2^CA^ mice were born in mendelian ratios and did not show any obvious gross morphological abnormalities. To investigate whether hematopoietic stem and progenitor cell (HSPC) pool was altered in IKK2^CA^ mice, we measured the frequencies of HPSCs in the BM. Adult murine HSPCs are identified based on their Lineage^-^Sca1^+^c-Kit^+^ (LSK) immunophenotype and contain both HSC and multipotent progenitor (MPP) fractions. Recent studies identified that HSC pool in the BM of mice contains at least two physiologically distinct cell types; (1) long-term (LT) HSCs that are largely quiescent and with long-term capacities, and (2) short-term (ST) HSCs that are actively diving and with reduced long-term capacities (Kiel and Morrison, 2008; Orford and Scadden, 2008; Trumpp et al., 2010; Pietras et al., 2011). Analysis of HSPC pool in the BM of IKK2^CA^ mice indicated an increase in the percentage (4.7% in control vs. 14.4% in IKK2ca mice) of LSK cells (Figure 1C). Distinguishing LSK cells into LT-HSCs (CD150^+^CD48^-^LSK) and MPPs (CD150^-^CD48^-^LSK) based on SLAM markers identified a decrease of both LT-HSCs (3.9% in control vs. 0.24% in IKK2^CA^ mice) and SLAM-MPPs (9.7% in control vs. 1.6% in IKK2ca mice) (Figure 1C). Similarly, dividing LSK cells into ST-HSCs (CD34^+^Flt3^-^LSK) and MPPs (CD34^+^Flt3^+^LSK) based on Flt3 and CD34 expression identified a relative increase (43% in control vs. 87% in IKK2^CA^ mice) of ST-HSCs and a decrease of conventional MPPs (49% in control vs. 9.5% in IKK2^CA^ mice) (Figure 1D). Determination of relative and absolute numbers indicated an increase of LSK cells (Figure 1E), decrease of LT-HSCs (Figure 1F), increase of ST-HSCs (Figure 1G) and decrease of MPPs (Figures 1H,I). These data indicated that constitutive activation of NF-κB was sufficient to cause reduction in the LT-HSC pool and to alter the distribution of HSPC numbers.

### Normal Numbers of Lineage Committed Progenitors in IKK2^CA^ Mice

To test whether constitutive activation of NF-κB signals impair differentiation of lineage committed progenitors of the myeloid and lymphoid lineage, we performed immunophenotyping studies. Analysis of progenitor compartments revealed comparable relative and absolute frequencies of common myeloid progenitors (CMPs; Lin^-^c-Kit^+^CD34^+^CD16/32^-^), granulocyte monocyte progenitors (GMPs; Lin^-^c-Kit^+^CD34^+^CD16/32^+^) and megakaryocyte erythrocyte progenitors (MEPs; Lin^-^c-Kit^+^CD34^-^CD16/32^-^) between IKK2^CA^ and control littermates (Supplementary Figures 1A,B). Similarly, lymphoid progenitor analysis indicated comparable relative and absolute numbers of common lymphoid progenitors (CLPs; Lin^-^c-Kit^med^Sca1^med^IL7Rα^+^) between control and IKK2^CA^ mice (Supplementary Figures 1C–E). To confirm these results, we performed an alternative surface staining that identifies CLPs based on Lin^-^c-Kit^med^Sca1^med^IL7Rα^+^Flt3^+^ immunophenotype, and found comparable numbers of CLPs between control and IKK2^CA^ mice (Supplementary Figure 1F). Similarly, lymphoid primed multi-potent progenitor (LMPPs; Lin^-^c-Kit^+^Sca1^+^Flt3^+^CD34^+^IL7Rα^+^) analysis did not reveal any difference between control and IKK2^CA^ mice (Supplementary Figure 1G). Consistent with normal distribution of lineage restricted progenitors, differentiation of myeloid and lymphoid lineage cells was relatively normal in IKK2^CA^ mice, although a modest decrease of B cells was noticed in the BM of these mice (data not shown). These data suggest that, even though the frequencies of MPPs were reduced due to constitutive NF-κB activation, differentiation of MPPs into CMPs, GMPs, MEPs, and CLPs was not impaired, at least, under steady state conditions.

### Defective Repopulation Capacities of HSCs From IKK2^CA^ Mice

**FIGURE 2:**
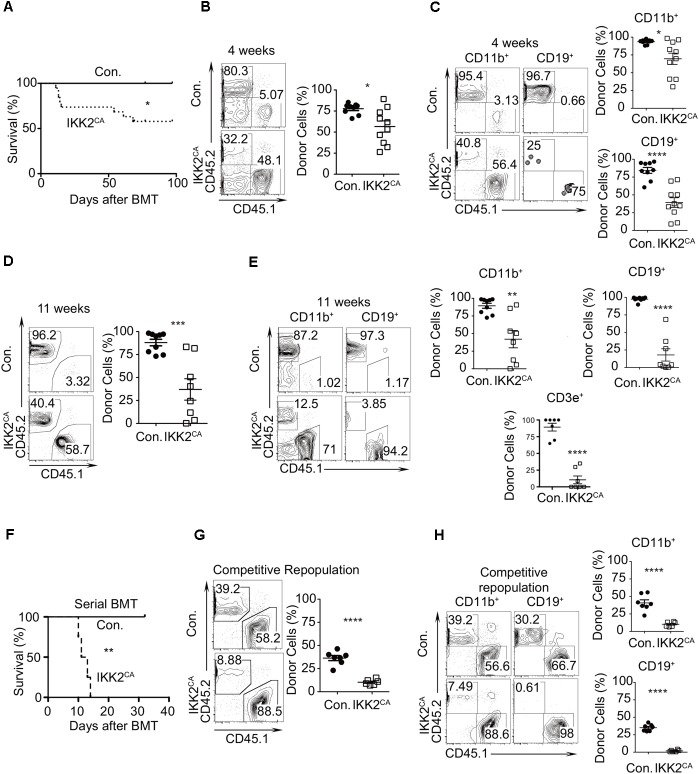
Loss of HSC quiescence in IKK2^CA^ mice. **(A)** Kaplan–Meier survival curve analysis of WT recipients (CD45.1^+^) that received BM of either IKK2^CA^ (*n* = 19) or control (*n* = 8) mice (CD45.2^+^) following lethal (10 Gy) irradiation. Significance (*P* = 0.0398) was assessed using the log-rank test. **(B)** Frequencies of donor (CD45.2^+^) derived hematopoiesis, at 4 weeks of transplantation, in the PB of WT (CD45.1^+^) recipients. Shown are representative FACS plots (left) and average frequencies (right). Data are pool of 9 mice in control group and 10 mice in IKK2^CA^ group. **(C)** Frequencies of donor (CD45.2^+^) derived CD11b^+^ myeloid cells and CD19^+^ B cells in the PB of WT (CD45.1^+^) recipients at 4 weeks of transplantation. Shown are representative FACS plots (left) and average frequencies (right). Data are pool of 9 mice in control group and 10 mice in IKK2^CA^ group. **(D)** Frequencies of donor (CD45.2^+^) derived hematopoiesis, at 11 weeks of transplantation, in the PB of WT (CD45.1^+^) recipients. Shown are representative FACS plots (left) and average frequencies (right). Data are pool of 9 mice in control group and 8 mice in IKK2^CA^ group. **(E)** Frequencies of donor (CD45.2^+^) derived CD11b^+^ myeloid cells, CD19^+^ B cells and CD3e^+^ T cells in the PB of WT (CD45.1^+^) recipients at 11 weeks of transplantation. Shown are representative FACS plots (left) and average frequencies (right). Data are pool of 9 mice in control group and 8 mice in IKK2^CA^ group. **(F)** Serial transplantation experiments: Kaplan–Meier survival curve analysis of secondary WT recipients (CD45.1^+^) that received BM of the primary recipients, following lethal (10 Gy) irradiation. Each group represents data from a pool of four animals. Significance (*P* = 0.0067) was assessed using the log-rank test. **(G)** Competitive repopulation assays: frequencies of donor (CD45.2^+^) derived hematopoiesis, at 4 weeks of transplantation, in the PB of WT (CD45.1^+^) recipients. Recipients received mixed chimera containing donor (CD45.2^+^) BM cells (from either IKK2^CA^ or control mice) and WT competitor (CD45.1^+^) BM cells at a ratio of 1:1. Shown are representative FACS plots (left) and average frequencies (right). Data are pool of 7 mice in control group and 6 mice in IKK2^CA^ group. **(H)** Competitive repopulation assays: frequencies of donor (CD45.2^+^) derived hematopoiesis, at 4 weeks of transplantation, in the CD11b^+^ myeloid cells and CD19^+^ B cells in PB of WT (CD45.1^+^) recipients. Shown are representative FACS plots (left) and average frequencies (right). Data are pool of 7 mice in control group and 6 mice in IKK2^CA^ group. All data represent mean ± SEM. Two-tailed Student’s *t*-tests were used to assess statistical significance (^∗^*P* < 0.05, ^∗∗^*P* < 0.01, ^∗∗∗^*P* < 0.001, ^∗∗∗∗^*P* < 0.0001). For survival curve analysis, log rank test was used to assess statistical significance (^∗^*P* < 0.05, ^∗∗^*P* < 0.01).

To evaluate whether the functions of HSCs were intact in IKK2^CA^ mice, we first performed bone marrow transplantation studies. Total BM cells (1 × 10^6^) from either control or IKK2^CA^ mice were injected *i.v.* into lethally (10 Gy) irradiated congenic (CD45.1^+^) wild type (WT) recipients. Strikingly, survival curve analysis indicated that ∼30% of the recipients, that received IKK2^CA^ BM, died within 3 weeks of transplantation and further analysis at longer time points suggested that only ∼50% of IKK2^CA^ BM recipients survived after 12 weeks of transplantation (Figure 2A). Whereas recipients that received control BM showed an average donor (CD45.2^+^) derived chimera of >70–80% in the peripheral blood at 4 weeks of transplantation, recipients that received IKK2^CA^ BM exhibited reduced donor derived chimera, with an average chimera of ∼50% (Figure 2B). Donor derived multi-lineage analysis indicated that both myeloid and lymphoid differentiation was compromised in IKK2^CA^ recipients, even though donor derived B lineage differentiation was more affected than myeloid differentiation (Figure 2C). Consistent with decreased donor derived hematopoiesis at 4 weeks, analysis of chimera at 11 weeks revealed a more severe reduction of IKK2^CA^ derived hematopoiesis (Figure 2D) and multi-lineage analysis suggested that majority of these donor cells were of myeloid lineage (Figure 2E). Interestingly, our analysis indicated that a few IKK2^CA^ recipient mice had a donor chimera of >75%, even after 11 weeks of transplantation (Figure 2D). However, transplantation of these BM cells from primary recipients into lethally irradiated secondary recipients failed to provide radioprotection (Figure 2F), suggesting that the BM of IKK2^CA^ recipients with high donor chimera was devoid of functionally competent HSCs.

Next, we tested the repopulation capacities of IKK2^CA^ BM cells under competitive settings. BM cells from either control or IKK2^CA^ mice (CD45.2^+^) were mixed with the BM cells of WT congenic (CD45.1^+^) mice at equal proportions (1:1) and were injected into lethally (10 Gy) irradiated WT congenic (CD45.1^+^) animals. While recipients of the control group showed an average of ∼40% of donor (CD45.2^+^) derived hematopoiesis, recipients that received IKK2^CA^ BM showed an average chimera of only ∼8%, at 4 weeks of transplantation (Figure 2G). Multi-lineage analysis indicated that both myeloid and lymphoid differentiation was affected under competitive conditions, however, donor derived B cell differentiation was absent in IKK2^CA^ recipients (Figure 2H). Together, these results indicated that constitutive activation of NF-κB results in impaired HSC functions.

### Augmented Proliferation and Diminished Quiescence of IKK2^CA^ HSCs

**FIGURE 3:**
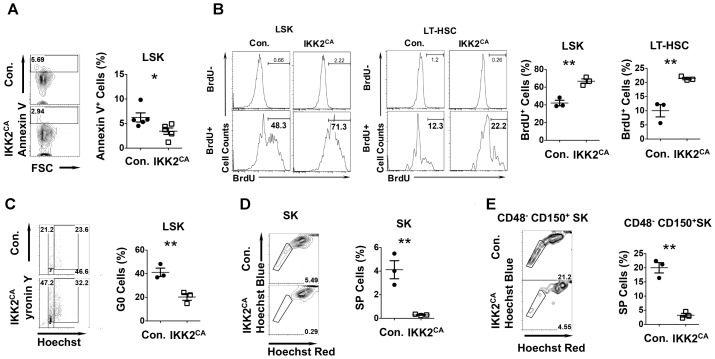
Altered cell cycle properties of HSCs from IKK2^CA^ mice. **(A)** Representative FACS plots (left) and average frequencies (right) of AnnexinV^+^ cells in LSK fractions in the BM of 6 weeks old IKK2^CA^ and control mice. Data are pool of 5 mice per group. **(B)** Representative FACS plots (left) and average frequencies (right) of BrdU^+^ cells in LSK cells and LT-HSCs in the BM of 6 weeks old IKK2^CA^ and control mice. Data are representative of two independent experiments with 3 mice per group. **(C)** Representative FACS plots (left) indicating frequencies of HSPCs in G0 (Pyronin Y^-^ and Hoechst^-^), G1 (Pyronin Y^+^ and Hoechst^-^) and G2/S (Pyronin Y^+^ and Hoechst^+^) phase of cell cycle in the BM of 6 weeks old IKK2^CA^ and control mice. Shown are average frequencies of HSPCs in G0 phase of cell cycle in the BM of 6 weeks old IKK2^CA^ and control mice (right). Data are representative of two independent experiments with 3 mice per group. Representative FACS plots (left) and average frequencies (right) of side population (SP) fraction in Sca1^+^c-Kit^+^ (SK) cells **(D)** and CD48^-^CD150^+^Sca1^+^c-Kit^+^ cells **(E)** in the BM of 6 weeks old IKK2^CA^ and control mice. Data are representative of two independent experiments with 3 mice per group. All data represent mean ± SEM. Two-tailed Student’s *t*-tests were used to assess statistical significance (^∗^*P* < 0.05, ^∗∗^*P* < 0.01).

To determine whether the loss of LT-HSC pool in the BM of IKK2^CA^ mice was due to increased apoptosis, we measured the frequencies of Annexin V^+^ cells in the LSK fraction. Surprisingly, the percentages of Annexin V^+^ cells were lesser in IKK2^CA^ mice, when compared to the control animals (Figure 3A). Next, we tested whether the proliferation kinetics of HSCs was altered in IKK2^CA^ mice. Our analysis indicated that the frequencies of BrdU^+^ cells were increased in both LSK and LT-HSC fractions (Figure 3B).

Based on these results we hypothesized that constitutive activation of NF-κB impairs HSC quiescence. To validate this, we first measured the frequencies of HSPCs in G0 phase under steady state conditions by using a combination of an RNA dye -pyronin and a DNA dye-Hoechst. Consistent with our hypothesis, we observed 46% of control LSK cells in the G0 phase, whereas only 14% of LSK cells from IKK2^CA^ mice was in G0 phase (Figure 3C). To further investigate HSC quiescence in IKK2^CA^ mice, we performed ‘side population’ (SP) studies, SP cells are a quiescent HSC population of immature LSK cells in the bone marrow (Arai et al., 2004). Strikingly, the data of SP studies identified 5% of quiescent cells in the Sca1^+^c-Kit^+^ BM fraction of control mice, while only 0.29% quiescent cells could be found in the same fraction of IKK2^CA^ mice (Figure 3D). Further studies identified 21% of the cells to be quiescent in the LT-HSC enriched CD48^-^CD150^+^Sca1^+^c-Kit^+^ BM fraction of control mice, whereas only 4% of the same was found to be quiescent in IKK2^CA^ mice (Figure 3E). These data strongly indicated that constitutive activation of NF-κB signals causes hyper-proliferation of HSCs and loss of quiescence in HSCs.

### Loss of ‘Stemness’ Signature in IKK2^CA^ HSCs

**FIGURE 4:**
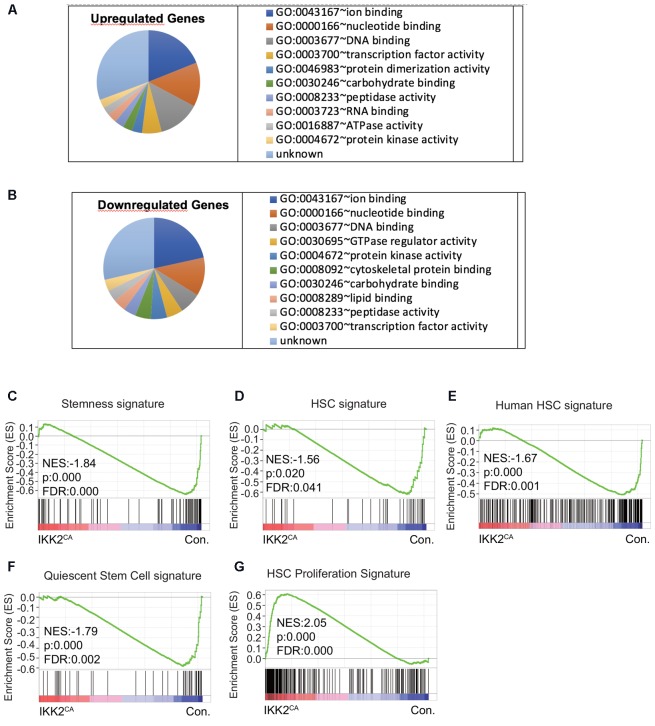
Deregulated molecular signatures in IKK2^CA^ HSCs. Gene ontology (GO) analysis of microarray data: top 10 major GO terms of molecular function among upregulated genes (>1.5 fold) **(A)** and downregulated genes (<0.67 fold) **(B)** in IKK2^CA^ LT-HSCs. Gene set enrichment analysis (GSEA) of microarray data from IKK2^CA^ versus control LT-HSCs with the following gene sets: genes enriched in normal, steady-state BM HSCs from WT mice compared to multi potent progenitors (MPPs), leukemic stem cells (LSCs), and mobilized HSCs **(C)**. Genes enriched in HSCs, but not in MPPs **(D)**. Genes enriched in human HSCs of cord blood **(E)**. Quiescent stem cell genes, which are commonly upregulated in HSCs, muscle stem cells and hair follicle stem cells **(F)**. Genes enriched in proliferative HSCs, which are commonly upregulated in fetal liver HSCs (vs. adult HSCs) and 2, 3, and 6 days after 5FU injection (vs. 0, 1, 10, 30 days after 5FU injection) **(G)**.

To uncover the molecular mechanisms through which constitutive NF-κB signals impair HSC quiescence and functions, we performed whole-genome transcriptome analysis. To this end, total RNA from CD150^+^ CD48^-^LSK cells from the BM of IKK2^CA^ and control mice was hybridized on to the Illumina MouseWG-6 v2.0 Expression BeadChips. A direct comparison of gene expression profiles between the control and IKK2^CA^ groups identified an upregulation (>1.5 fold) of 475 genes and a downregulation (<0.67 fold) of 672 genes in LT-HSCs of IKK2^CA^ mice (Supplementary Table 1). Next, we performed gene ontology (GO) analysis, to get an overview on the major functional alterations that are caused by constitutive NF-κB signaling in HSCs. Data revealed that the major molecular functions of the upregulated genes (Figure 4A) in IKK2^CA^ LT-HSCs include; ion binding, DNA binding, nucleotide binding, and TF activity. Similarly, the major molecular functions of the downregulated genes (Figure 4B) in IKK2^CA^ LT-HSCs include; ion binding, nucleotide binding, DNA binding, and TF activity. Interestingly, the functions of 115 upregulated genes and of 166 downregulated genes in IKK2^CA^ LT-HSCs remain unknown.

To gain further insights into the molecular identities of the gene expression programs that are differentially expressed in IKK2^CA^ LT-HSCs, we performed GSEA (Subramanian et al., 2005), as this can provide information about gene clusters shared by the IKK2^CA^ LT-HSCs signature and publicly available data sets. Accordingly, we first compared our gene profiling data with the published HSC ‘stemness’ signature data sets. These data sets were identified as genes enriched in normal, steady-state BM HSCs, when compared to MPP, LSC, and mobilized HSC (Forsberg et al., 2010). Our analysis revealed that the HSC ‘stemness’ signature was almost lost in IKK2^CA^ LT-HSCs (Figure 4C). To corroborate these results, independently, we compared our gene array data with the published (Park et al., 2002) HSC enriched-data sets that were identified as genes highly enriched in HSCs, but not in MPPs. As expected, these results confirmed that the HSC signature was abolished in IKK2^CA^ LT-HSCs (Figure 4D). Furthermore, a direct comparison of our expression profiling data with the data sets generated from human HSCs enriched gene sets, revealed a down regulation of human HSC signature in IKK2^CA^ LT-HSCs (Figure 4E). These data suggest that constant NF-κB signals are disadvantageous to the molecular regulatory circuits of HSCs.

### Impaired ‘Quiescence’ Signature in IKK2^CA^ HSCs

One of the major cellular mechanisms by which HSC ‘stemness’ and functions are regulated is through induction of quiescence (Morrison and Weissman, 1994; Passegue et al., 2005; Wilson et al., 2008; Trumpp et al., 2010). Our analayis revealed that HSC quiescence is impaired in IKK2^CA^ mice. Thus, we compared our gene expression data with the quiescence-related data sets (Cheung and Rando, 2013), that were identified as ‘quiescence’ signature that is common in HSCs, muscle stem cells and hair follicle stem cells. In line with our experimental data (Figure 3), this GSEA indicated that the HSC ‘quiescence’ signature was lost in IKK2^CA^ LT-HSCs (Figure 4F). Consistent with lack of “quiescence” signature, comparison of our data with published proliferative HSC-related data sets (Venezia et al., 2004) identified an enrichment of “proliferation” signature in IKK2^CA^ LT-HSCs (Figure 4G).

**FIGURE 5:**
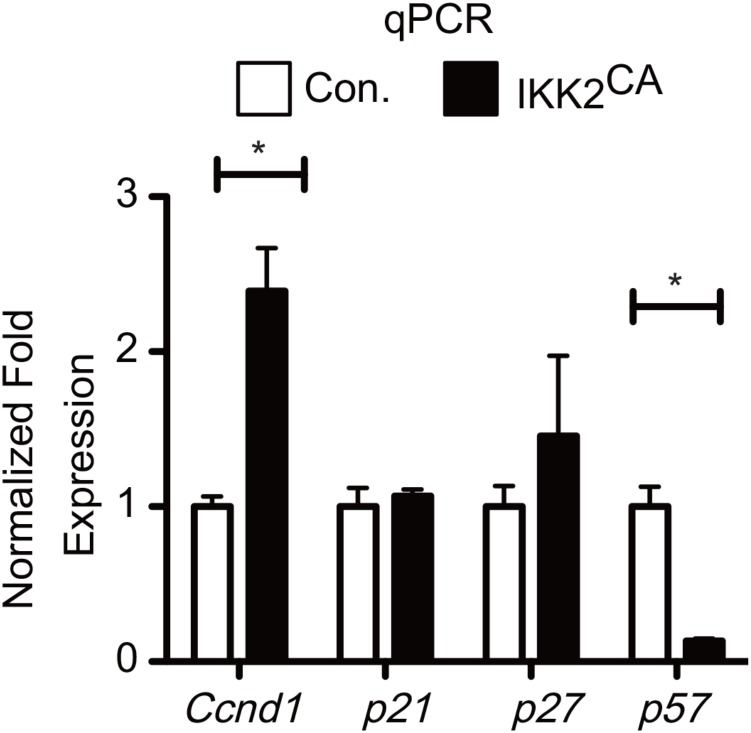
Deregulated gene expression levels of Cell cycle regulators in IKK2^CA^ HSCs. Real time PCR data for *Ccnd1, p21, p27*, and *p57* in IKK2^CA^ and Control LT-HSCs. Expression levels of target genes were normalized to HPRT levels. Data are representative of two independent experiments. All data represent mean ± SEM. Two-tailed Student’s *t*-tests were used to assess statistical significance (^∗^*P* < 0.05).

To explore the molecular mechanisms through which NF-κB signals suppress HSC quiescence, we directly focused on the expression of key genes that may influence cell cycle. Pioneering studies have unequivocally proven that combined actions of both cyclin dependent kinases (CDKs) and cyclin dependent kinase inhibitors (CDKIs) regulate HSC quiescence (Orford and Scadden, 2008; Pietras et al., 2011). To obtain insights into the deregulated molecular mechanisms that might affect HSC quiescence in IKK2^CA^ mice, we analyzed the expression levels of positive regulators of cell cycle, such as *Ccnd1, Ccnd2, Ccnd3, Cdk4* and *Cdk6*, and negative regulators of cell cycle, such as *p15^ink4b^, p16^ink4a^, p18^ink4c^, p19^ink4d^, p19^arf^, p27^kip^*, and *p57^kip2^* in IKK2^CA^ LT-HSCs. To validate the microarray data through an independent technique, we performed real-time PCR analysis. As expected, expression levels of *Ccnd1* were increased and of *p57^kip2^* were reduced in IKK2^CA^ LT-HSCs (Figure 5), even though the fold differences between control and IKK2 mutant LT-HSCs were more striking from the data of real-time PCR assays. Of note, loss of D-cyclins in HSCs results in increased quiescence (Pietras et al., 2011) and a deficiency of p57 causes loss of HSC quiescence and impaired reconstitution of the hematopoietic system (Matsumoto et al., 2011), a phenotype that is very similar to IKK2^CA^ HSCs. These data from GSEA and real-time PCR analysis provide a molecular explanation for impaired HSC quiescence in IKK2^CA^ mice and indicate that the HSC ‘quiescence’ signature is sensitive to constitutive NF-κB signals.

### Deregulated ‘Transcription Factor Networks’ in IKK2 Mutant HSCs

**FIGURE 6:**
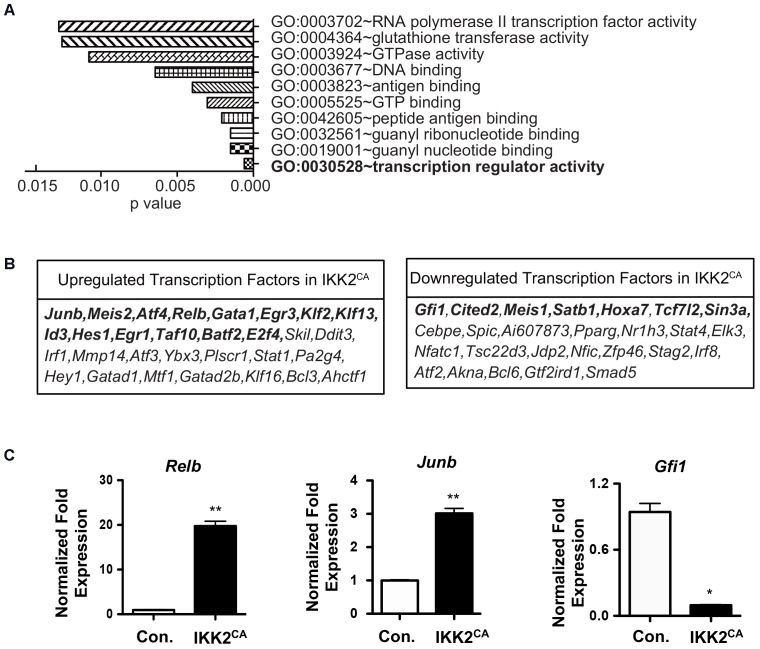
Altered transcription factor networks in IKK2^CA^ HSCs. **(A)** Gene ontology analysis of microarray data: the 10 most significantly enriched GO terms (function) in upregulated genes (>1.5 fold) in IKK2^CA^ LT-HSCs. *P*-values were calculated by DAVID 6.7 software. **(B)** Differentially expressed transcription factors (TFs) in IKK2^CA^ LT-HSCs. Highlighted TFs have been shown to regulate HSC self-renewal, quiescence, and differentiation. **(C)** Real time PCR data for *Relb, Junb*, and *Gfi1* in IKK2^CA^ or Control LT-HSCs. Expression levels of target genes were normalized to HPRT levels. Data are representative of two independent experiments. All data represent mean ± SEM. Two-tailed Student’s *t*-tests were used to assess statistical significance (^∗^*P* < 0.05 and ^∗∗^*P* < 0.005).

Combinatorial actions, that include antagonistic or cooperative effects, of TFs play indispensable roles in the control of self-renewal, differentiation, and functions of HSCs (Orkin, 2000). Based on our *in vivo* and *in silico* findings, we hypothesized that a tight control on NF-κB signaling is critical to maintain the ‘stemness’ associated ‘TF networks’ in HSCs. Indeed, a more refined GO analysis on the list of upregulated genes identified changes in transcription regulator activity (GO:0030528) as the major and most significant (*P* = 0.0006) functional consequence caused by increased NF-κB activity in LT-HSCs (Figure 6A and Supplementary Table 2). Overall, our analysis identified a differential expression of 56 (30 upregulated and 26 downregulated) TFs in IKK2^CA^ LT-HSCs (Figure 6B). To further understand the TF networks that were deregulated by constitutive NF-κB signals in HSCs, we focused on the specific functions of these TFs. Our analysis on upregulated TFs indicated that positive regulators of HSC differentiation; *Relb, Gata1, Egr3, Klf2, Klf13, Id3, Hes1, Egr1, Taf10, Batf2* and *E2f4*, and negative regulators of HSC self-renewal and functions; *Junb, Meis2*, and *Atf4* were upregulated in the IKK2 mutant LT-HSCs (Figure 6B). On the other hand, TFs that positively regulate HSC self-renewal, quiescence and functions; *Gfi1, Cited2, Meis1, Satb1, Hoxa7, Tcf7l2*, and *Sin3a* were downregulated in the LT-HSCs of IKK2^CA^ mice (Figure 6B). To validate the expression levels of selected TFs through an independent technique, we performed real-time PCR analysis. In agreement with the microarray data, results of real-time PCR experiment suggested an upregulation of *Junb* and *Relb*, and a downregulation of *Gfi1* (Figure 6C). In keeping with our results, increased expression of *Junb* (Passegue et al., 2004) and reduced expression of *Gfi1* (Hock et al., 2004) are associated with loss of self-renewal, quiescence, and functions of HSCs. Overall, these data indicated that aberrant activation of NF-κB pathways in HSCs affects its physiology by; (1) inducing expression of multiple TFs associated with lineage differentiation and (2) repressing expression of key TFs that contributes to HSC self-renewal and functions. These results are of unique importance, as it suggests that constitutive activation of NF-κB is sufficient to alter the regulatory circuits of TFs that are critical to HSC self-renewal and functions.

### Augmented NF-κB Binding to *Junb* in IKK2^CA^ Hematopoietic Cells

**FIGURE 7:**
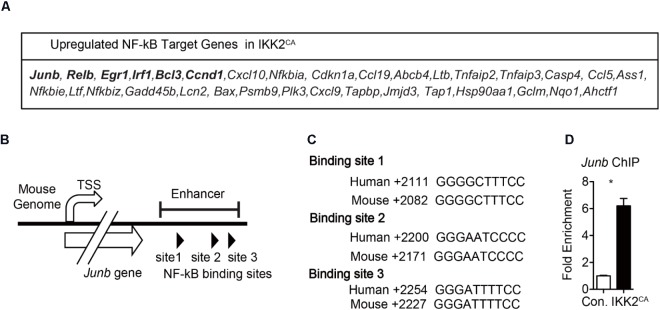
Increased NF-κB binding to *Junb* enhancer region in hematopoietic cells from IKK2^CA^ mice. **(A)** NF-κB target genes that are upregulated in IKK2^CA^ LT-HSCs. **(B)** Diagrammatic representation of the *Junb* gene indicating the presence of three NF-κB binding sites in its enhancer region. **(C)** NF-κB binding sites in enhancer region of the *Junb* gene of indicated species. **(D)** Chromatin immunoprecipitation (ChIP) analysis of NF-κB (p65) binding to *Junb* promoter in the hematopoietic cells of 6 weeks old IKK2^CA^ and control mice. Shown are the Real Time PCR data of p65 immunoprecipitates, that were normalized to IgG control immunoprecipitates. Data are representative of two independent experiments. All data represent mean ± SEM. Two-tailed Student’s *t*-tests were used to assess statistical significance (^∗^*P* < 0.05, ^∗∗^*P* < 0.01).

NF-κB family of proteins functions as TFs by binding to the RelA binding sites found in the regulatory regions of the target genes (Ghosh and Hayden, 2008; Vallabhapurapu and Karin, 2009). While NF-κB proteins have been documented to bind and activate over 500 target genes, their key transcriptional targets in HSCs remain largely unknown. In an attempt to identify the direct targets of NF-κB in HSCs, we compared the list of differentially genes in IKK2^CA^ HSCs with the public database that contains the list of validated NF-κB target genes. This analysis indicated an upregulation of 33 potential NF-κB target genes in IKK2 mutant LT-HSCs (Figure 7A). In general, NF-κB proteins are believed to act as transcriptional activators, rather than transcriptional repressors, upon binding to the regulatory regions of their target genes (Ghosh and Hayden, 2008; Vallabhapurapu and Karin, 2009). Hence, we focused exclusively on the upregulated NF-κB targets in IKK2^CA^ LT-HSCs, as these genes may represent the key targets of NF-κB in HSCs. Among the 33 candidate genes, we found upregulation of five TFs; *Junb, Relb, Egr1, Irf1*, and *Bcl3.* Of these, *Junb* caught our immediate attention for at least two following reasons; (1) *Junb* is the most abundantly expressed TF in IKK2^CA^ LT-HSCs and (2) Overexpression of *Junb* led to the loss of quiescence and functions of HSCs (Passegue et al., 2004). Next, we explored if increased transcription of *Junb* was a directly associated with increased NF-κB binding in IKK2^CA^ hematopoietic cells. To this end, we followed the ‘*in silico*’ approach to identify the presence of NF-κB binding site(s) in the regulatory regions of *Junb.* Our analysis identified three NF-κB binding sites in the enhancer region (+2028 to +2249) of *Junb (*Figure 7B) and all these three NF-κB binding sites are well-conserved between mouse and humans (Figure 7C). Of note, previous studies highlighted the importance of this enhancer region in NF-κB mediated regulation of *Junb* expression (Salem et al., 2013). To check if constitutive activation of NF-κB results in augmented NF-κB binding to its target sites in the enhancer region of *Junb*, we performed ChIP assays. In line with our hypothesis, data of ChIP experiments documented an increase in binding of NF-κB to the enhancer region of *Junb* (Figure 7D). It is noteworthy that the HSC phenotype of IKK2^CA^ mice is very similar to the phenotype of *Junb* overexpressing HSCs (Passegue et al., 2004). Even though our data suggest that *Junb* is overexpressed in IKK2^CA^ HSCs due to increased NF-κB binding to its enhancer region, we believe that deregulated expression of *Junb* may not be wholly responsible for the defective HSC functions in IKK2^CA^ mice. Based on our gene expression and bioinformatics studies we hypothesize that uncontrolled NF-κB activity introduces several alterations at a genetic and molecular level, which in turn may be responsible for the impaired HSC quiescence and functions.

## Discussion

In the present study, we investigated the impact of persistent IKK2/NF-κB activation in HSC maintenance and functions. Data reveal that constitutive IKK2/NF-κB activation causes many alterations in the HSPC compartments, such as depletion of the LT-HSC pool, increased numbers of ST-HSCs and reduction in MPP numbers. The decrease of LT-HSCs was not due to increased apoptosis, instead, LT-HSCs of IKK2^CA^ mice showed augmented proliferation and reduced quiescence. A potential explanation for this could be that these LT-HSCs were proliferating more and subsequently differentiating into ST-HSCs, without undergoing self-renewal divisions. However, it was interesting that even though the numbers of ST-HSCs were augmented, the frequencies of MPPs were reduced in IKK2^CA^ mice. This might be due to either a block in the differentiation of ST-HSCs into MPPs or increased differentiation of MPPs into lineage committed progenitors. However, committed progenitors of both myeloid and lymphoid lineages did not show any significant differences between control and constitutively active IKK2 mice. Together, these data suggest that the HSPC fraction, but not lineage committed progenitor faction, is more sensitive to constitutive activation of IKK2.

At a molecular level, the loss of HSC quiescence and defective HSC functions observed in constitutively active IKK2 mice could be explained, at least partially, by the loss of CDKI- p57 expression. Earlier studies identified p57 as a key factor of HSC quiescence and maintenance (Matsumoto et al., 2011). According to this report, p57 deficient HSCs show reduced self-renewal, decreased proportion HSCs in the G0 phase, decreased colony forming unit (CFUs) and defective hematopoietic reconstitution abilities (Matsumoto et al., 2011). Thus, the HSC phenotype of IKK2^CA^ mice is very similar to that of p57 deficient mice. Even though our studies established an interesting link between constitutive activation of IKK2/NF-κB pathway and deregulated p57 expression, understanding the precise molecular mechanisms through which IKK2/NF-κB signaling suppress p57 expression would be an exciting area of future investigation.

In the current study, we document that increased IKK2 activation leads to deregulated expression of TF networks in HSCs. Based on earlier investigations, on the role of TFs in hematopoiesis (Shivdasani and Orkin, 1996; Orkin, 2000; Zhu and Emerson, 2002; Iwasaki et al., 2006; Chen et al., 2014), and our own studies documenting increased binding of NF-κB to the promoters of its target genes; IFN-γ (Nakagawa et al., 2015) and *Junb* (Figure 7), we speculate that the HSC phenotype of IKK2^CA^ mice is caused, primarily, due to increased binding of NF-κB to its targets in HSCs. However, we cannot rule out the involvement of additional mechanisms that might indirectly influence the physiology of HSCs. Of note, in the current studies we utilized Vav^cre^ transgenic mice to activate NF-κB in HSCs. Even though Vav^cre^ has been used extensively in studies involving HSCs and considered as a faithful cre line to induce recombination of floxed alleles in HSCs and in their progeny, Vav^cre^ mouse strain has also been reported to generate recombination in vascular endothelial cells (Georgiades et al., 2002), albeit at lower efficiencies. Our analysis of IKK2CA Vav^cre^ mice indicated an absence of recombination in endothelial lineage cells of the BM (data not shown). However, we cannot rule out other possibilities through which endothelial cells (Poulos et al., 2016) might affect HSCs of IKK2^CA^ mice, especially during the fetal hematopoiesis. Furthermore, in IKK2^CA^Vav^cre^ mice NF-κB is constitutively active in differentiated hematopoietic lineage cells, including neutrophils, macrophages, dendritic cells, and NK cells. In view of the fact that NF-κB controls expression of inflammatory cytokines in these immune cells and that inflammatory cytokines can directly influence the physiology of HSCs, including quiescence (Essers et al., 2009; Sato et al., 2009; Baldridge et al., 2010), it remains possible that the HSC phenotype of IKK2^CA^Vav^cre^ mice might be influenced, at least in part, by these non-cell intrinsic mechanisms. A future study with a focus on delineating the involvement of cell intrinsic vs. extrinsic pathways of NF-κB mediated regulation of HSC physiology would be essential in understanding the precise role of NF-κB in hematopoiesis.

In the recent years, the importance of non-canonical NF-κB signals in early hematopoietic events has begun to unfold to a greater extent (Vallabhapurapu and Karin, 2009; Gerondakis et al., 2012; Zhao et al., 2012; González-Murillo et al., 2015; Xiu et al., 2017). However, to date, there are only a few studies that described the role of canonical NF-κB signals in HSC biology. Grossmann et al. (1999) reported that the combined loss of *c-Rel* and *RelA* did not affect pluripotent stem cells. However, this study did not analyze the ‘bona fide’ HSC compartment and their functions (Grossmann et al., 1999). Stein and Baldwin described that a deficiency of RelA resulted in impaired HSC functions, increased proliferation and reduced apoptosis of HSPCs (Stein and Baldwin, 2013). Taken together, these studies have highlighted that loss of NF-κB signals results in increased HSC cycling and impaired HSC functions. Based on these previous findings, it may be expected that increased NF-κB signaling would promote quiescence and functions of HSCs. However, surprisingly, our data indicate that constitutive activation of NF-κB signals in HSCs causes diminished HSC quiescence and functions. It is interesting to observe that the HSC phenotype of IKK2^CA^ mice is similar to the HSC phenotype of RelA^-/-^ mice. Even though right now it is unclear, as why and how both loss of NF-κB signals and gain of NF-κB signals would cause a similar phenotype, these data suggest that a ‘fine tuning’ of NF-κB signals is critical to HSC biology. More mechanistic studies would be essential to unravel the functional complexities executed by NF-κB in HSCs.

In essence, the study presented here explored the functional consequences of constitutive activation of NF-κB in HSCs and attempted to unravel the molecular mechanisms and through which NF-κB controls HSCs. Our data identify that deregulated NF-κB signals lead to loss of HSC quiescence and functions. Furthermore, these results indicate that increased NF-κB signaling perturbs the TFs networks in HSCs. A clearer understanding on the precise role of NF-κB in normal and pathologic hematopoiesis may provide new therapeutic opportunities.

## Author Contributions

MN performed the research, collected and interpreted the data, and prepared the figures. HC performed the research and collected the data. CR designed the research, interpreted data, and wrote the manuscript.

## Conflict of Interest Statement

The authors declare that the research was conducted in the absence of any commercial or financial relationships that could be construed as a potential conflict of interest.

## Supplementary Material

FIGURE S1 Differentiation of lineage committed progenitors remains intact in IKK2^CA^ mice.

TABLE S1 Differentially expressed genes in IKK2CA/+ HSCs.

TABLE S2 Gene ontology (GO) analysis on upregulated genes in IKK2CA/+ HSCs.
