# Supplementary figures and images for: Constitutive Activation of NF-κB Pathway in Hematopoietic Stem Cells Causes Loss of Quiescence and Deregulated Transcription Factor Networks
Source: Front Cell Dev Biol. 2018 Oct 30;6:143. doi: 10.3389/fcell.2018.00143 (PMC6218573; doi:10.3389/fcell.2018.00143)

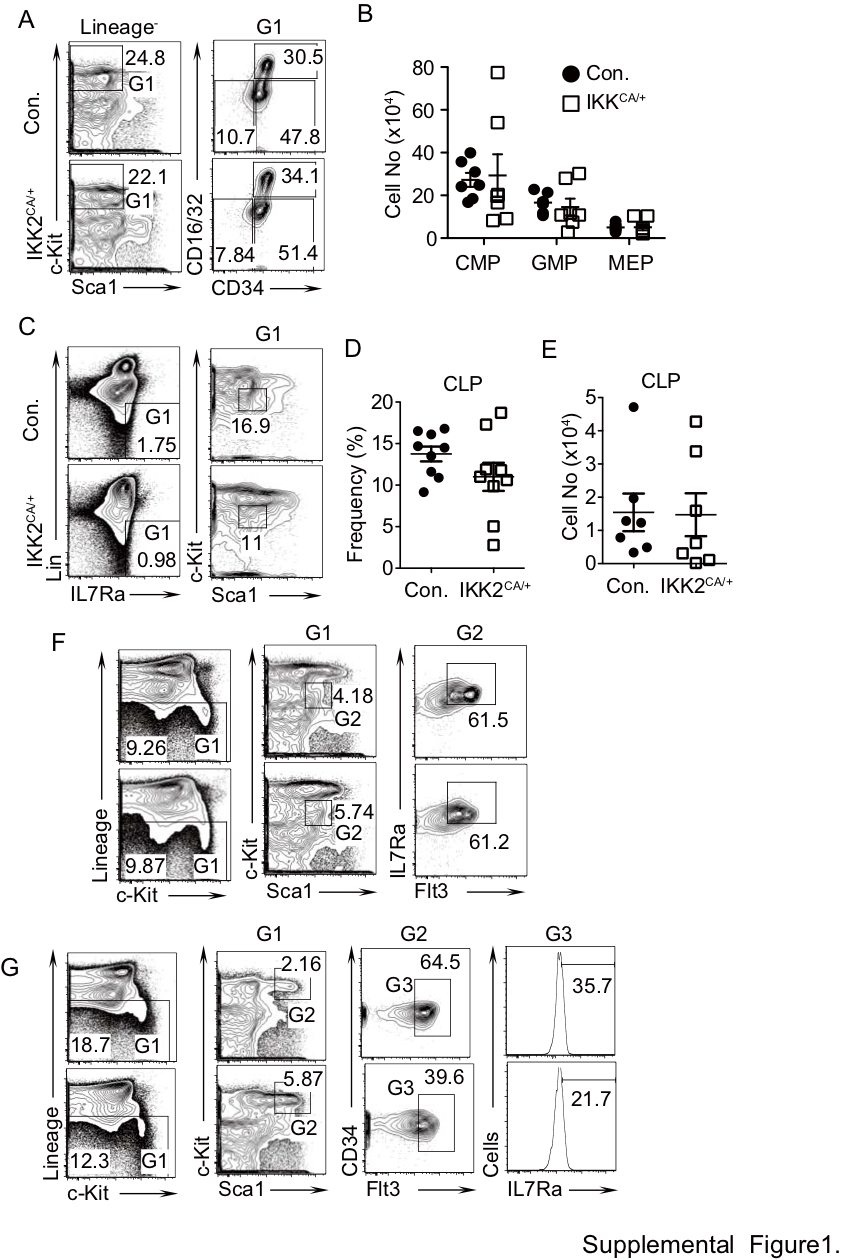

Supplement: FIGURE S1 — Differentiation of lineage committed progenitors remains intact in IKK2CA mice. [file Image_1.TIFF]
